# Pharmacological enhancement of memory or cognition in normal subjects

**DOI:** 10.3389/fnsys.2014.00090

**Published:** 2014-05-20

**Authors:** Gary Lynch, Conor D. Cox, Christine M. Gall

**Affiliations:** ^1^Department of Psychiatry and Human Behavior, University of CaliforniaIrvine, CA, USA; ^2^Department of Anatomy and Neurobiology, University of CaliforniaIrvine, CA, USA

**Keywords:** cognitive enhancement, learning, long term potentiation, ampakine, synaptic plasticity, BDNF, F-actin, positive AMPA receptor modulators

## Abstract

The possibility of expanding memory or cognitive capabilities above the levels in high functioning individuals is a topic of intense discussion among scientists and in society at large. The majority of animal studies use behavioral endpoint measures; this has produced valuable information but limited predictability for human outcomes. Accordingly, several groups are pursuing a complementary strategy with treatments targeting synaptic events associated with memory encoding or forebrain network operations. Transcription and translation figure prominently in substrate work directed at enhancement. Notably, the question of why new proteins would be needed for a now-forming memory given that learning-driven synthesis presumably occurred throughout the immediate past has been largely ignored. Despite this conceptual problem, and some controversy, recent studies have reinvigorated the idea that selective gene manipulation is a plausible route to enhancement. Efforts to improve memory by facilitating synaptic encoding of information have also progressed, in part due of breakthroughs on mechanisms that stabilize learning-related, long-term potentiation (LTP). These advances point to a reductionistic hypothesis for a diversity of experimental results on enhancement, and identify under-explored possibilities. Cognitive enhancement remains an elusive goal, in part due to the difficulty of defining the target. The popular view of cognition as a collection of definable computations seems to miss the fluid, integrative process experienced by high functioning individuals. The neurobiological approach obviates these psychological issues to directly test the consequences of improving throughput in networks underlying higher order behaviors. The few relevant studies testing drugs that selectively promote excitatory transmission indicate that it is possible to expand cortical networks engaged by complex tasks and that this is accompanied by capabilities not found in normal animals.

## Introduction

The present review concerns three topics, two of which involve terms—enhancement and cognition—that are not sharply defined. Usage of the former seems straightforward when applied to memory, although it is often unclear whether accelerated acquisition or an increase in encoding strength is intended. But applied to cognition, claims for enhancement face the great problem of how to quantify something for which there is no consensus measurement system. The difficulty can be reduced by focusing on cognitive activities of a type that can be described in computational terms. This, however, raises questions about the extent to which the sampled process is representative, or a major component, of cognition as the term is typically used. In response, it could reasonably be argued that cognition is a collection of semi-independent operations (e.g., categorization, value assignment) (Sugrue et al., 2005; Tsunada and Sawaguchi, 2012) but this seems unsatisfactory because the phenomenon is experienced as being, if not unitary, then at least strongly coherent. Electrophysiological and brain imaging results showing coordinated activity across broad stretches of neocortex provide some support for the idea of a system that, while capable of periodically focusing on specific tasks, usually works by integrating a vast amount of disparate material into a product accessible to consciousness. A true cognitive enhancer might therefore take the form of a treatment that increases the speed or capacity of this assembly process.

Memory enhancement, as suggested, appears to be a much more tractable problem. Retention is easily measured as is the amount of training needed to produce a given score in a test subsequent to learning. But a curious problem emerges here: few of the many pharmacological agents that produce robust enhancement of memory in animals are found to have positive effects in humans. This observation has become the subject of intense public discussion, perhaps with growing skepticism about the utility of animal studies on memory enhancement. Some neuroscientists have argued that the “failure to predict” problem reflects the widespread use of paradigms that have little relevance to human learning. These workers have devised ingenious protocols that can be used in rodents and with minor modifications in humans (e.g., Bari et al., 2008; Demeter et al., 2008; Eichenbaum and Robitsek, 2009; Zeeb et al., 2009; Demeter and Sarter, 2013). There is every reason to assume that these efforts will ultimately narrow the gap in cross-species comparisons. But there is a more fundamental issue from comparative biology that could underlie the failure-to-predict problem: humans are enormously encephalized animals and rodents aren't (neocortex makes up at least 77% of brain volume in human and just 31% in rat; Stephan et al., 1981; Swanson, 1995). Encephalization is hypothesized to result in a shift of functions from lower brain to cortex; from this perspective, humans may be using networks of a very different kind than those employed by rodents to solve similar problems.

An alternative to behaviorally based approaches to developing enhancers would be to focus on the neurobiological substrates of memory and cognition. This seems feasible in the case of memory because of the tremendous progress that has been made in identifying synaptic mechanisms that encode information. There is no good reason to think that these processes differ significantly between mammalian species and indeed comparative studies suggest that certain essential elements are evolutionarily ancient (Crystal and Glanzman, 2013). It follows from this that treatments acting on memory substrates in rodents are likely to have similar actions in human brain. Cognition again represents a much more challenging problem. However, the universally held assumption that cognitive operations arise from the transient formation of telencephalic networks points to a relatively simple idea for enhancement. Communication within and between cortical regions is mediated by glutamatergic transmission; if so, then agents that augment the release of glutamate, or the post-synaptic response to it, should facilitate the formation of cognition's substrates.

The following sections consider attempts to develop enhancers via actions on (i) different aspects of the complex machinery underlying learning-related synaptic modifications, or (ii) communication within and between cortical networks.

## Memory enhancement

Most research on memory enhancement deals with psychological events that precede the actual encoding of information. There is for example a very large literature describing attempts, typically using chemical agents, to increase the speed of learning by modulating arousal and attention (Lynch et al., 2011). It has become common to refer to resultant improvements as cognitive enhancement, presumably because key elements of cognition are being manipulated, but there are reasons to question this assumption (see below). There is a smaller, but rapidly growing, body of work directed at the machinery responsible for converting patterns of afferent activity into the long lasting increases in synaptic strength assumed to encode specific information. This section evaluates the latter material.

### Gene expression and protein synthesis

Work in this area begins with the hypothesis that learning triggers the transcription or local translation of proteins that serve to consolidate the newly acquired memories, something that can take anywhere from many minutes to hours. Compounds that facilitate production of the pertinent RNAs or proteins could accordingly increase the likelihood that recent learning will lead to stable memory, and there are many reports of such effects (Guzowski et al., 2001; Plath et al., 2006; Katche et al., 2010, 2012). However, the basic idea that new protein synthesis is critical to memory formation has been controversial since its introduction more than 50 years ago (Abraham and Williams, 2008; Gold, 2008). Much of the dispute revolves around the necessary prediction that protein synthesis inhibitors will selectively block recently acquired memory; most papers report this result but others do not, or argue that observed disruptions to encoding are due to factors unrelated to synthesis (Routtenberg, 2008; Gold and Wrenn, 2012).

Beyond this, the protein synthesis argument faces certain conceptual problems. Learning is a continuous process in humans, and likely other mammals, with new encoding occurring many times a minute, as is evident with episodic memory. People recognize or recall a remarkable number of serial events when queried after a 90 min movie. Unless we make the very unlikely assumption that each item of information is encoded on a different neuron, it is difficult to see why, after hours of producing proteins needed for consolidation, a given cell would need further synthesis to stabilize a now forming memory. Along this line, it has been argued that animals exposed to an enriched environment which would entail constitutively elevated basal activity, and thus activity-driven protein synthesis, may not require additional synthesis to support LTP (Abraham and Williams, 2008) and the related encoding of hippocampus-dependent memories. There is, however, a special case in which transcription and/or broadly distributed translation could be required to securely encode a specific memory; namely, a circumstance in which continuous learning of similar material does *not* precede the new instance. Under these conditions, consolidation could depend upon proteins generated by the isolated learning episode. Note that this scenario loosely describes the great majority of animal studies testing for the contributions of protein synthesis. Certain of these arguments make relatively straightforward, readily tested predictions. For example, animals with a well-developed learning set could be given protein synthesis inhibitors after learning a single problem with or without having dealt with many such problems in the preceding hours. Such a paradigm can be achieved for rats using two-odor discriminations. If continual learning obviates the need for problem-specific synthesis, then the blockers should have no effect in a group given many trials prior to being introduced to the new test items.

There is a variant of the translation hypothesis that addresses the problem of why prior synthesis doesn't provide a sufficient supply of proteins for current learning. This involves the ample evidence for dendritic (local) translation from already in place mRNAs. One could posit a set of conditions in which new synthesis, even after recent experience, needs to occur post-acquisition for transfer into long-term storage; e.g., (1) translation occurs within very small dendritic compartments; (2) such active regions are only found in the immediate vicinity of recently modified synapses; and (3) newly formed proteins do not diffuse to any great degree. These circumstances would reduce the probability that proteins from earlier learning would be present at the large majority of current sites. But “synaptic tagging” experiments, conducted for instances where LTP in hippocampal slices is blocked by protein synthesis inhibitors, describe results that are not consistent with these postulates. Specifically, LTP induction at one input protects subsequently induced potentiation at a second input to the same region from the effects of the inhibitor (Frey and Morris, 1997; Shires et al., 2012). Given the small number of synapses that generate EPSPs of conventional amplitudes, it is extremely likely that connections from the two inputs are, for the most part, located on different dendritic segments. It follows then that proteins from the first episode must have been synthesized, or traveled, throughout much of the dendritic arborization, a point that is reinforced by evidence for tagging in the apical dendrites after stimulation of basal afferents (Alarcon et al., 2006). It will be noted that these findings align with the broad idea that continual learning maintains relevant proteins at levels sufficient for LTP-related plasticity, obviating the need for synthesis after individual learning events.

The above discussion concerns interpretative issues rather than the likelihood of achieving enhancement using the transcription / translation strategy. It may well be the case that increasing within-cell levels of proteins that support consolidation reduces the requirements for encoding persistent memories and/or increases their stability. Signaling from synapses to the nucleus or to local protein synthesis machinery involves many steps and so is likely to be a variable and somewhat uncertain process. It would not be surprising, then, if the ongoing production of memory-related elements operates at a less than optimal rate even in high performing, normal subjects. In line with this, there are multiple demonstrations that treatment with compounds that inhibit particular histone deacetylases, leading to increased transcription of select gene families, can markedly enhance memory after single training sessions (Stefanko et al., 2009; McQuown et al., 2011). Also of interest are the numerous studies showing that selective phosphodiesterase-4 inhibitors have potent enhancing effects on memory. Inhibitors of this class (e.g., Rolipram), drive the protein kinase A—CREB transcription pathway implicated in learning in a broad array of animals (including invertebrates), and so is argued to be a very ancient, evolutionarily conserved memory substrate (Tully et al., 2003; Normann and Berger, 2008). Evidence that the same results obtain after extensive experience with similar problems in the recent past, and presumably a great deal of learning-driven transcription, would constitute support for there being less than optimal production of proteins needed for encoding under normal circumstances. This would certainly encourage the idea that enhanced protein synthesis is a viable route to augmented memory.

### Synaptic plasticity and memory enhancement

**Figure 1:**
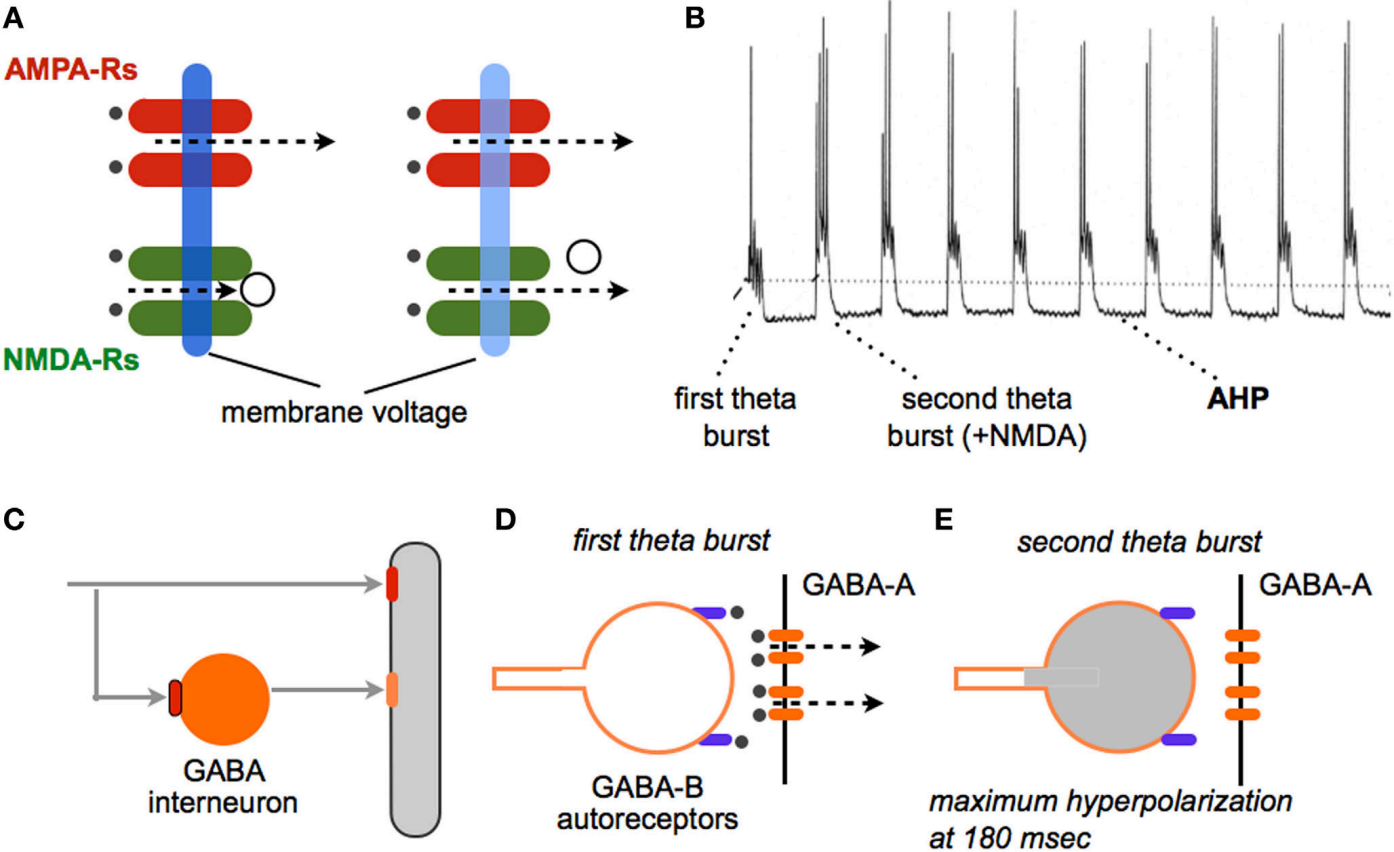
**Why theta burst stimulation (TBS) is so effective at inducing LTP**. TBS (Larson et al., 1986) mimics a firing pattern found in cortical neurons during learning (Otto et al., 1991) and elicits a robust, non-decremental LTP that persists for weeks (at least). **(A)** (left side). A single stimulation pulse releases glutamate (black dots) and a partial membrane depolarization via current flux through AMPA receptors (dotted line). NMDA receptors do not open because of voltage dependent block of the ion channel (open circle). (right side) Trains of high frequency stimulation cause a greater depolarization (light blue) that removes the channel block and thereby allows current flow through calcium permeant NMDA receptors. Calcium is the initial trigger for LTP. **(B)** Intracellular recording shows that the first theta burst (four pulses at 100 Hz) in a train causes a relatively modest depolarization accompanied by a single spike; NMDA receptors make a very small contribution to this response. A second burst administered after a delay corresponding to the period of the theta wave produces a more profound depolarization with multiple spikes; this burst response contains a large NMDA receptor mediated component. Note that each theta burst in the train is followed by a large after-hyperpolarization (AHP). The AHP, which is largely mediated by calcium and voltage dependent potassium channels, tends to counteract the depolarization produced by the burst, thus capping the magnitude of NMDA receptor responses. **(C–E)** How the second and subsequent bursts generate large depolarization and unblock NMDA receptors. **(C)** A glutamatergic axon innervates a pyramidal cell dendrite (gray) and a feedforward, GABAergic interneuron (orange); note that *both* contacts use AMPA receptors (red). **(D)** A first theta burst triggers GABA release from the interneuron onto the pyramidal neuron thereby producing a di-synaptic (slightly delayed) IPSC via post-synaptic GABA-A receptors (orange ellipses); this shunts the EPSCs produced at neighboring glutamatergic synapses. The released GABA also binds to pre-synaptic, metabotrophic GABA-B auto-receptors on the releasing interneuron terminal (purple). **(E)** The auto-receptors hyperpolarize the GABAergic terminal and block release, an effect that reaches its maximum at the period of the theta wave. A theta burst arriving at this time point generates an excitatory response that is only weakly counteracted by the opening of post-synaptic GABA-A receptors (see **B**).

Most mechanism-based efforts directed at improving memory have focused on synaptic plasticity and in particular the long term potentiation (LTP) effect. Researchers since the late 19th century have argued that the enormous capacity of memory is best explained by assuming that physical encoding of new information occurs at small numbers of connections between neurons. The discovery of LTP demonstrated that individual synapses in the cortical telencephalon do in fact possess the properties expected for a memory substrate (Bliss and Collingridge, 1993; Lynch, 1998, 2004b; Morris, 2003). The increase in transmission strength (magnitude of EPSCs) develops quickly, persists for a remarkable period (weeks at least) (Staubli and Lynch, 1987; Abraham, 2003), and does not disturb already potentiated contacts as likely required for a high capacity memory system. A very large body of experimental work has confirmed the tight connection between LTP and diverse instances of memory (e.g., Roman et al., 1987; Rioult-Pedotti et al., 2000; Whitlock et al., 2006). Moreover, LTP is intimately related to the theta rhythm, an oscillation long associated with learning (Buzsaki, 2005; Vertes, 2005; Snider et al., 2013); i.e., five brief (30 ms) bursts of high frequency stimulation pulses (a pattern that mimics “theta bursting” during learning) prove to be near optimal for inducing extremely stable LTP but only when separated by the period of the theta wave (Larson et al., 1986; Capocchi et al., 1992). The reasons for this have been identified (Figure 1).

These observations suggest the possibility of enhancing learning with drugs that promote theta activity and correlated bursts of high frequency discharges. Agents such as physostigmine, that facilitate central cholinergic transmission, promote the theta rhythm (Olpe et al., 1987; Hasselmo, 2006) and are reported to improve learning scores in certain experimental situations. Notably, drugs of this type are among the few treatments approved for Alzheimer's Disease (Clarke and Francis, 2005; Noetzli and Eap, 2013). However, cholinergic systems perform varied functions in brain, some of which are homeostatic in nature. This likely explains why drugs targeting cholinergic mechanisms have not gained widespread acceptance as plausible enhancers. Another approach based on theta activity involves the large hyperpolarizing potentials triggered within target neurons by the short train of theta bursts used to induce LTP. These after-hyperpolarizing potentials (AHPs), set in motion by cell discharges, persist throughout the duration of the theta train and serve to counteract the depolarization needed to unblock the voltage dependent, synaptic NMDA receptors. Influx of calcium through these receptors, followed by release of the cation from intracellular stores, triggers the chain of events leading to potentiation (Figure 1). AHPs are mediated by a set of voltage- and calcium-sensitive potassium channels, prominent among which is the SK3 channel (Hosseini et al., 2001). The bee toxin apamin blocks this channel with some selectivity and, as predicted, augments post-synaptic responses to theta burst trains; this results in a striking increase in the magnitude of LTP (Kramar et al., 2004). While a number of studies have found substantial improvements in rodent learning with apamin treatment (Ikonen and Riekkinen, 1999; Brennan et al., 2008; Vick et al., 2010), this is not a likely enhancer because of toxicology issues. But given increasing interest in applications of channel blockers for diverse clinical problems, the apamin results suggest an intriguing mechanistic target for the development of enhancers. It is of note in this regard that Brain Derived Neurotrophic Factor (BDNF), which appears to be released from terminals by theta bursts (Balkowiec and Katz, 2000; Chen et al., 2010b), also reduces AHPs at least in rats (Kramar et al., 2004). Elevating endogenous levels of this neurotrophin, which can be achieved by pharmacological manipulations described later, thus provides another avenue for enhancement.

Identification of the initial triggers for LTP, as schematized in Figure 1, pointed to NMDA receptor-mediated calcium influxes as a logical target for enhancement. The existence of multiple modulatory sites (e.g., for glycine and polyamine) on the receptors suggested a plausible route for building positive allosteric drugs (Monaghan et al., 2012). Most of this effort has been directed toward treatments for neuropathology and psychiatric disorders, most notably schizophrenia and depression (Labrie and Roder, 2010; Dang et al., 2014), rather than memory enhancement. Perhaps the most widely studied agent of this type is D-cycloserine, a compound that targets the glycine binding pocket on the receptor and facilitates channel opening (Sheinin et al., 2001; Dravid et al., 2010). It has been known for some time that the site is important for induction of LTP (Oliver et al., 1990) and, as expected from this, D-cycloserine enhances various forms of memory in animals (Flood et al., 1992; Baxter et al., 1994; Tsai et al., 1999; Normann and Berger, 2008; Peters and De Vries, 2013). There is also evidence that the endogenous neurosteroid pregnenolone sulfate (Wu et al., 1991), and other steroid-like substances (Madau et al., 2009), promote the opening of NMDA receptors and facilitate both LTP and memory. Also of note, recent work led to discovery of a naturally occurring cholesterol metabolite that facilitates NMDA receptor currents through a novel oxysterol modulatory site and markedly increases the magnitude of LTP (Paul et al., 2013). The development of positive NMDA receptor modulators is clearly a promising area with regard to enhancement.

**Figure 2:**
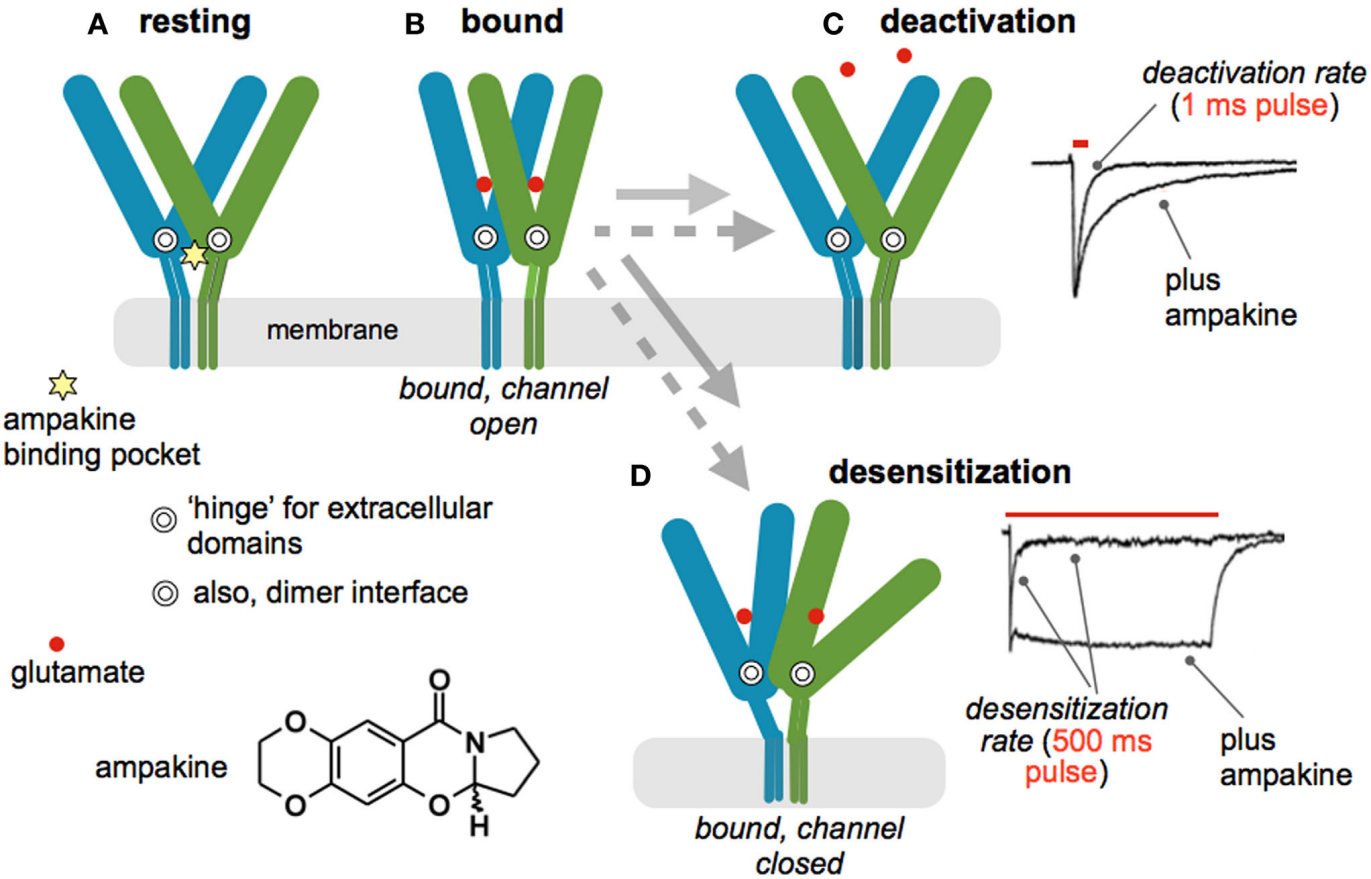
**Mode of action for positive allosteric modulators of AMPA receptors (ampakines). (A)** Schematic shows two of the four subunits that comprise the AMPA receptor tetramer in the resting state; the C-tails are not included. Each subunit has two large extracellular domains that form a “clamshell” containing the glutamate binding site. The hinge of the structure is indicated by the double circle. The subunits dimerize at a zone close to the hinge. The ampakine pocket (star) is strategically located at the dimer interface adjacent to the two hinges. There are thus four neurotransmitter, and two ampakine, sites on the full AMPA receptor. **(B)** Glutamate binding is accompanied by a closing of each subunits' clamshell, resulting in opening of the ion channel and inward flux of current. The receptor then shifts into one of two configurations; the gray arrows denote the time required for the transitions in the presence (dotted) and absence (solid line) of an ampakine. **(C)** Normally, single transmission events are followed by opening of the extracellular domains and release of the transmitter, a process referred to as “deactivation.” The upper trace to the right describes deactivation after a one ms pulse of glutamate to an excised patch: delivery of the ligand causes a sharp influx of current that decays after rapid washout. Bound ampakines slow reopening, resulting in a significant retardation of deactivation (bottom trace). **(D)** Prolonged stimulation of the receptor can disrupt the dimer configuration, leading to a condition in which transmitter remains bound but the ion channel returns to the closed state (desensitization). The upper trace to the right describes an instance of this in which glutamate was applied for 500 msec. An initial influx of current was followed by decay, despite continuing presence of the transmitter, to a steady state value about 1/10 of the peak flux. Ampakines stabilize the dimer configuration and, as predicted, greatly slow desensitization—current flow continues throughout the 500 ms application of glutamate. The receptor structural dynamics, including interactions with an ampakine, illustrated here are based on X-ray crystallography studies (Sun et al., 2002; Jin et al., 2005); physiological data are from patches taken from hippocampal slices (Arai et al., 1996, 2002; Arai and Lynch, 1998).

Increasing current flux through AMPA receptors results in greater post-synaptic depolarization and thereby promotes removal of the voltage block on NMDA receptors. This suggests that increasing AMPA receptor currents should facilitate the induction of LTP. Tests of this became possible with the invention of AMPA receptor modulators that freely enter the brain and increase fast glutamatergic transmission (Lynch, 2004a). The initial positive modulators were small benzamide compounds but subsequent work from many laboratories resulted in diverse families of compounds that slow deactivation or desensitization (or both) of ligand bound AMPA receptors. Here we will refer to all agents of this type by the term, “ampakines,” used for the original compounds. Through a series of electrophysiological and X-ray crystallography studies, the mechanism of ampakine action is now fairly well understood. As illustrated in Figure 2, each subunit of the tetrameric AMPA receptor has two large extracellular domains that form a “clamshell” that closes upon glutamate binding (Sun et al., 2002). Relaxation to the resting state, and transmitter release, terminates current flow; this process is referred to as “deactivation.” The four subunits form two dimers, an arrangement that can be disrupted by ligand binding; under these conditions the channel closes but the transmitter is retained. This interesting, high affinity (slow dissociation constant) state constitutes the desensitized condition of the receptor (Hall et al., 1993). It was originally thought that desensitization is the normal route for terminating the EPSC but it now appears that deactivation is responsible for the decay rate of the synaptic response. The ampakine binding pocket is located at the dimer interface near the hinge of the clamshell (Jin et al., 2005); this strategic position explains how ampakines can affect both deactivation and desensitization (Arai et al., 1996) (Figure 2). Apparently, the orientation of the compounds within the pocket determines which of the two processes is most affected. There is overlap between AMPA and NMDA receptor pharmacology: compounds widely used to block the former also exhibit high affinity antagonism of the glycine modulatory site on the latter (Kessler et al., 1989). However, the ampakine pocket is distant to the extracellular domain of AMPA receptor antagonist binding and there is no evidence that these drugs affect NMDA receptor-gated currents.

Early work established that ampakines enhance both LTP and memory (Granger et al., 1993; Staubli et al., 1994), results that have been multiply replicated by different groups (Lynch, 2004a; Lynch and Gall, 2013). Versions of the drugs that simply slow deactivation lower the threshold for inducing LTP whereas those that affect both deactivation and desensitization also raise the ceiling on the degree of potentiation produced by theta bursts (Arai et al., 2002). By changing rate constants for both receptor inactivation processes, the latter compounds lead to much longer EPSCs and thus prolonged NMDA receptor-mediated calcium influxes. This presumably explains their greater potency. Surprisingly, there appear to have been no studies testing for differential actions of the two functional classes of ampakine on learning.

Notably, the positive influence of acutely administered ampakines on memory are reported for aged and young animals (Granger et al., 1993, 1996; Shors et al., 1995) as well as for a broad array of species and learning tasks (Lynch, 2004a; Bernard et al., 2010). Very few effects in human have been published although one study using a short half-life, deactivation-only drug obtained evidence for memory enhancement in different tasks including ones involving complex processing (Ingvar et al., 1997).

### Learning-related synaptic modifications as a target for enhancement strategies

**Figure 3:**
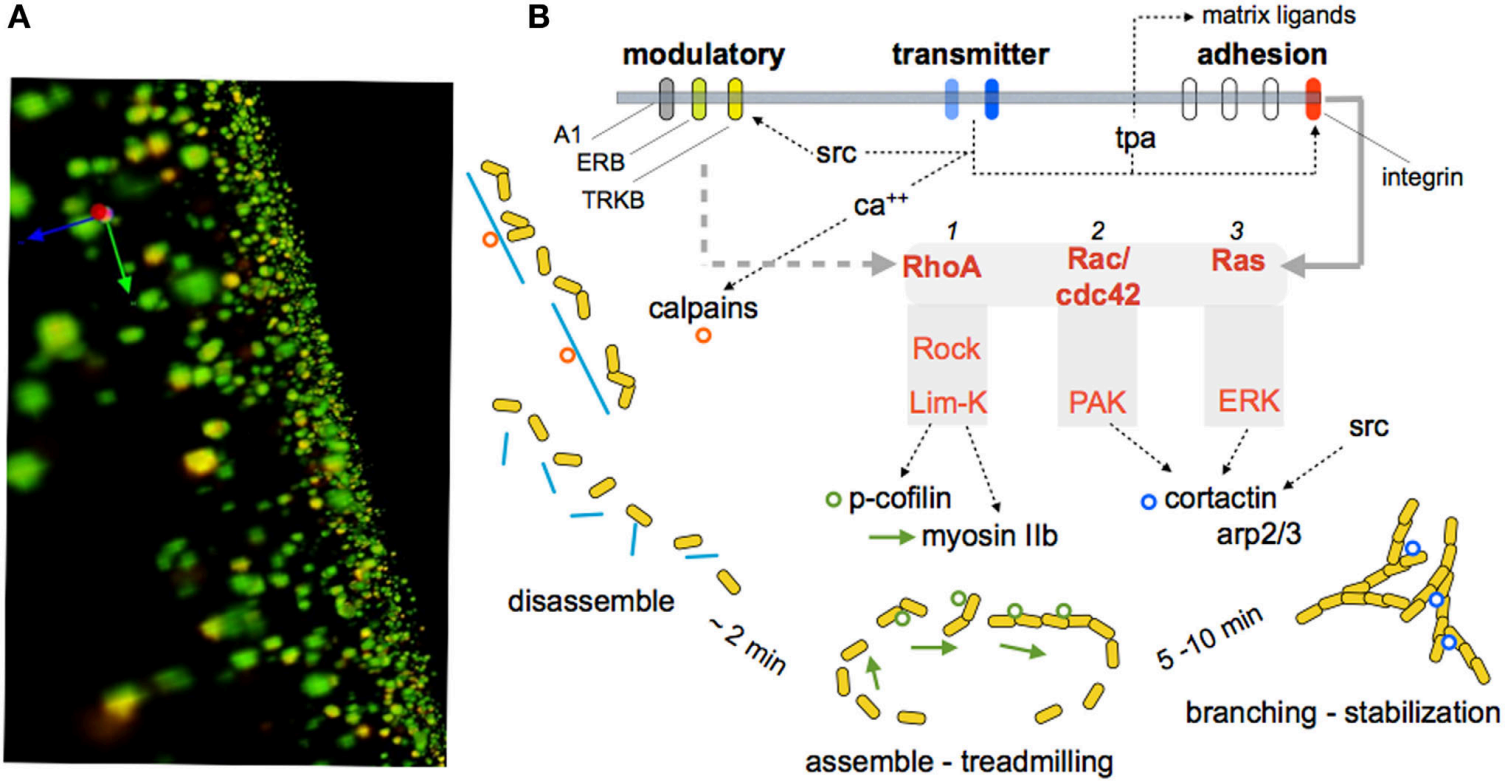
**Signaling events responsible for reorganizing the synaptic cytoskeleton and consolidating LTP**. The substrate map for LTP stabilization is largely based on work using hippocampal slices, although some of the steps have been observed in learning studies. **(A)** Immunolabeled synapses surrounding the LTP site as reconstructed using Fluorescence Deconvolution Tomography (Seese et al., 2013): The green elements reflect immunostaining for PSD95, a protein that is evenly distributed within post-synaptic densities at excitatory (glutamatergic) synapses. Phosphorylated (inactivated) cofilin was immunolabeled with red fluorescence. Co-localization (p-Cofilin/PSD95) results in yellow labeling. The technique supports counts and size-measures for about 40,000 synapses per image z-stack and 160,000 synapses per slice, and calculates the number of these synaptic elements that are co-localized with the signaling protein of interest (p-Cofilin in this instance). These values are then compared for slices that did or did not receive theta burst simulation. **(B)** Schematic shows signaling pathways activated at excitatory synapses by theta burst stimulation. Transmitter receptors i) increase calcium which stimulates calpain, a spine protease (Perlmutter et al., 1988) that cleaves cross-linking proteins (blue lines) for the subsynaptic cytoskeleton, and ii) activates synaptic adhesion receptors belonging to the integrin family (Babayan et al., 2012). Integrins then engage at least two Rho family GTPases that promote the assembly of dynamic actin filaments (RhoA to cofilin and myosin) (Rex et al., 2009) and, over a period of several minutes, branching and stabilization of the reorganized cytoskeleton. The latter processes involve Rac/Cdc42 signaling to cortactin and ARP2/3. The synaptic membrane also contains receptors for the releasable factors adenosine, estrogen, and BDNF (A1, ERB, TrkB, respectively). These receptors positively and negatively (A1) influence the signaling pathways, probably at the level of the GTPases. Studies using neutralizing antisera, genetic manipulations, toxins, and enzyme blockers confirm certain key links in the model and show that disrupting these specific actin regulatory pathways blocks the consolidation, but not initial expression, of LTP.

The discovery of LTP (Bliss and Lomo, 1973) greatly simplified what had already been an extended search for the substrates of memory. An early and critical clue came with electron microscopic evidence that stable potentiation is accompanied by changes in the morphology of dendritic spines (Lee et al., 1979, 1980; Chang and Greenough, 1984), an observation recently and convincingly confirmed by live imaging experiments (Matsuzaki et al., 2004; Harvey and Svoboda, 2007; Kramar et al., 2012b). The initial studies also described results suggestive of an increase in synapse size and there are now data pointing to a similar effect after LTP (Chen et al., 2007) and learning (Fedulov et al., 2007). The observed anatomical restructuring implied that induction events for LTP or memory result in substantial alterations to the actin cytoskeleton. Tests of this, using a newly developed *in situ* method for labeling F-actin in hippocampal slices, found that theta bursts cause a dramatic increase in the number of spines with high concentrations of polymerized actin (Lin et al., 2005; Kramar et al., 2006). The newly formed filaments proved to be unstable for a period of 5–10 min, after which they were unaffected by depolymerizing agents (Rex et al., 2009, 2010). The experimental question then became one of how the very brief AMPA and NMDA receptor events that induce LTP lead to the formation of new actin cytoskeleton. Work using Fluorescence Deconvolution Tomography for assessing concentrations of activated signaling proteins at individual synapses, along with the use of selective inhibitors, identified multiple, GTPase-initiated signaling pathways involved in the assembly and stabilization of actin filament networks following theta burst stimulation (Kramar et al., 2009; Rex et al., 2009, 2010; Seese et al., 2012). Particularly relevant to the present topic, these studies also described membrane receptors that modulate the activity of cascades leading to the cytoskeletal reorganization required for consolidation of LTP (Figure 3).

**Figure 4:**
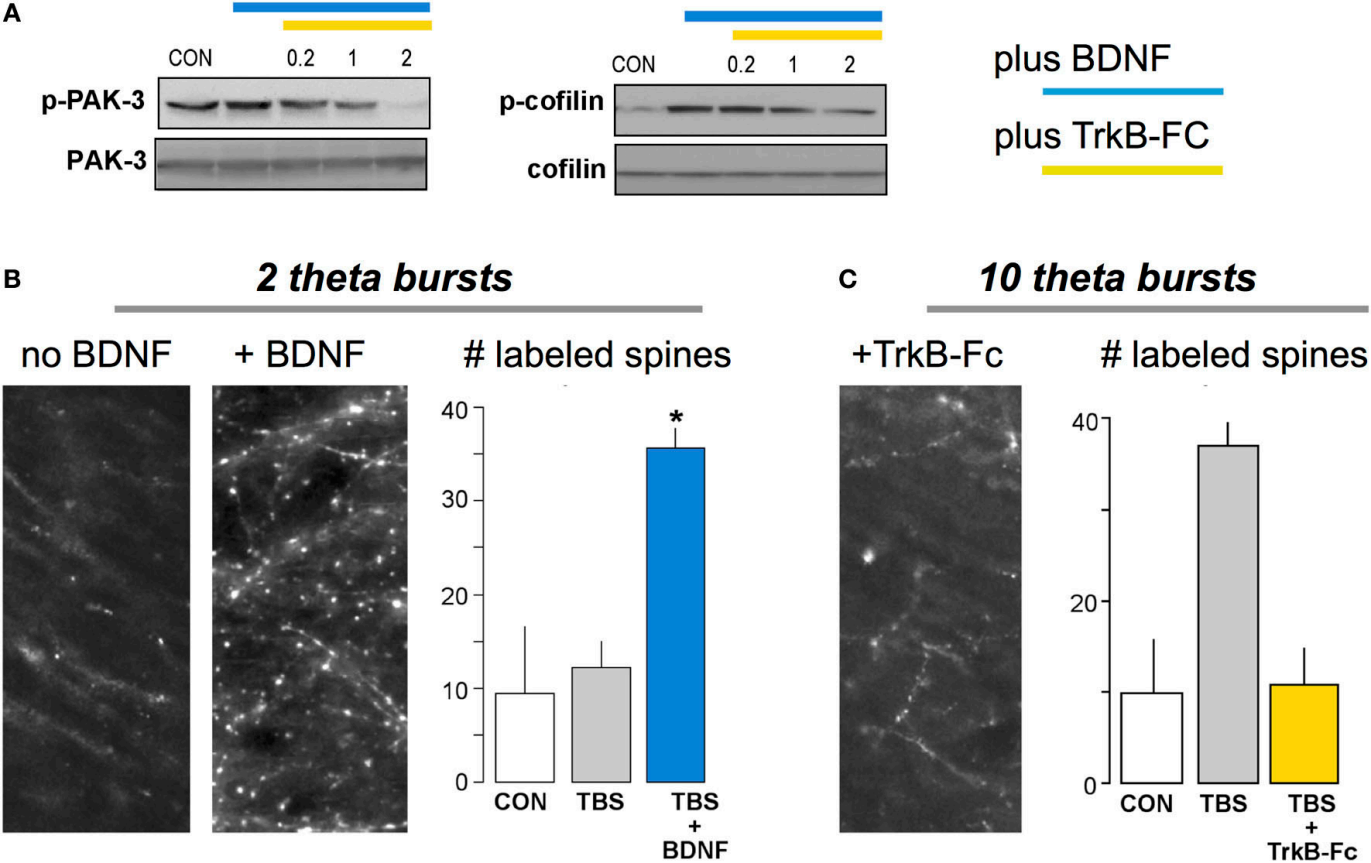
**BDNF enhances theta burst induced actin signaling and cytoskeletal assembly at hippocampal synapses. (A)** Brief infusion of BDNF (60 ng/ml) into adult hippocampal slices increases phosphorylation of PAK and cofilin (immunoblots), two of the signaling proteins included in the schematic for LTP consolidation (Figure 3). Phosphorylation was blocked by addition of the extracellular BDNF scavenger TrkB-Fc (applied at 0.2–2 μg/ml). There were no evident effects of either treatment on levels of total PAK or cofilin. **(B)** Labeling of filamentous (F-) actin with fluorescence-tagged phalloidin applied to slices 10 min after delivery of two theta bursts (TBS), a number too small to generate LTP if applied alone. Pretreatment with BDNF (right image) caused a marked increase in TBS-induced labeling of dendritic spines relative to labeling in ACSF-bathed slices receiving similar stimulation. Graph: Summary of the number of spines with intense concentrations of F-actin, as assessed using automated counting for a fixed sampling field, confirmed these observations (CON, slices received low frequency stimulation only; ^*^p < 0.01 vs. CON). **(C)** Similar to **(B)** except that ten theta bursts were used. Pretreatment with TrkB-Fc completely blocked the otherwise robust increase in F-actin positive spines seen with ten burst TBS. Modified from Rex et al. (2007).

Brief treatments with BDNF partially activate at least two of the signaling pathways shown in Figure 3 and potently facilitate both theta burst-driven actin polymerization and LTP (Chen et al., 2006; Rex et al., 2007). It seems likely that the LTP effects reflect both direct actions on the actin regulatory pathways and the above noted influence on AHPs generated during the theta stimulation trains (see above). Notably, scavenging extracellular BDNF blocks the stabilization of LTP produced by theta burst stimulation (Kovalchuk et al., 2002; Rex et al., 2007) as well as the associated activation of actin regulatory signaling and increases in spine F-actin (Figure 4); activity-induced release of the neurotrophic factor thus emerges as a key ingredient in the normal production of learning-related synaptic changes. In all, increases in BDNF signaling appear to be a biologically plausible means for enhancing memory. Peripheral administration of the protein is unlikely to have robust central effects but brain permeant agonists for its synaptic TrkB receptor have been developed and shown to improve function in varied conditions of impairment (Andero et al., 2012; Schmid et al., 2012; Ding et al., 2013; Jiang et al., 2013). Reports on how these compounds affect memory in normals have only begun to appear but initial studies indicate that acute systemic treatment can improve object recognition, object location and fear memory when given just before training (Andero et al., 2011; Bollen et al., 2013); for object location memory administration 3 h after training was also effective. These results encourage the expectation that acute systemic treatment with a TrkB agonist can facilitate both initial encoding and mechanisms of consolidation for at least some forms of memory. Further work is needed to determine the range of learning and cognitive functions that respond to this strategy and if this occurs without deleterious side effects.

Another route for utilizing BDNF in memory studies is suggested by the observation that transcription of the factor is positively regulated by neuronal activity (Isackson et al., 1991; Gall, 1992). It follows from this that increases in excitatory drive to neurons, as for example produced by ampakines, should up-regulate the neurotrophin. A sizable number of studies using individual or a series of daily injections of the positive modulators have confirmed this basic idea (Lauterborn et al., 2000; Rex et al., 2006; Simmons et al., 2009; Bernard et al., 2010; Haditsch et al., 2013). The treatments rescue theta burst-induced actin polymerization and LTP in a number of animal models of human conditions in which memory loss and/or intellectual disabilities are prominent, including those for normal aging, low estrogen levels, early stage Huntington Disease, and Angelman syndrome (Rex et al., 2006; Simmons et al., 2009; Baudry et al., 2012; Kramar et al., 2012a). When tested, daily injections also reduced or eliminated memory impairments (Simmons et al., 2009; Baudry et al., 2012). Several weeks of daily ampakine treatment were shown to be well tolerated. They also markedly reduced pathology and improved motor functioning in a mouse model of early onset Huntington Disease (Simmons et al., 2011); subsequent work with systemic administration of a TrkB agonist obtained similar results (Simmons et al., 2013).

Although it is apparent that semi-chronic ampakine treatment increases BDNF protein levels, and has potent brain effects predicted from this, there appear to be no studies testing for influences of up-regulating BDNF on learning in normal, high functioning animals. This likely reflects an assignment of greater importance to treatment than to enhancement with regard to drug development. But the exciting results obtained with up-regulation and receptor agonists with regard to brain disorders make BDNF-based strategies one of the more promising mechanism-grounded approaches to achieving memory enhancement.

The substrate map for LTP consolidation includes estrogen receptor beta as a second membrane agent that exerts a powerful modulatory influence over the actin signaling leading to LTP consolidation. Thirty minute infusions of estrogen, at physiological concentrations, cause a modest increase in baseline transmission in hippocampus but a striking facilitation of LTP (Cordoba Montoya and Carrer, 1997; Foy et al., 1999; Bi et al., 2000; Kramar et al., 2009). Recent work showed that these effects are due to activation of one of the actin regulatory cascades initiated by theta bursts (i.e., RhoA>ROCK>LIMK>cofilin—see Figure 3) and the assembly of new filamentous actin in spine heads (Kramar et al., 2009). Unlike the case for BDNF, there are several reports that estrogen improves memory scores in high functioning subjects across tasks and species (Frye et al., 2007; Liu et al., 2008; Hara et al., 2014). Evidence for similar effects in humans appears to be lacking (Grodstein, 2013) although several studies describe a decline in verbal memory with surgical menopause and improvements with hormone replacement (Brinton, 2009). Beyond needing further evidence for effects in cognitively normal individuals, a primary barrier to development of an estrogen-based enhancement strategy lies in the fact that the steroid affects many fundamental cellular processes in brain and the periphery, and is known to facilitate certain types of cancer. More restricted actions can be had using agonists selective for the hormone's beta receptor which is, to a degree, concentrated in brain; such agonists are highly effective in LTP studies (Kramar et al., 2009). Evidence that estrogen is synthesized by hippocampal neurons and that hormone of local origin contributes significantly to hippocampal synaptic plasticity (Ooishi et al., 2012; Vierk et al., 2012) should also be noted here. Thus, it may be possible to find means to promote normal, likely activity-dependent, estrogen actions in a regionally restricted manner.

### Integration: Many paths to the same end

Brain scientists had proposed increases in the strength of connections between neurons as the substrate of memory before the introduction of the word “synapse” (Cajal, 1894). The idea is intuitively attractive since such increases would clearly alter the operation of cortical networks and thus behavior. In essence, it describes microscopic events that, when implemented at many sites, could be the physical instantiation of the macroscopic phenomenon of memory. From this perspective, the most direct route to memory enhancement would involve facilitating physiologically produced, long lasting increases in synaptic responses. Developing what is still only an outline of the machinery that induces, expresses, and consolidates LTP then shaped ideas about how to produce facilitation. To some extent, it also led to a unification that is perhaps under-appreciated: an unrelated array of enhancement candidates such as steroids, trophic factors, positive modulators of glutamate receptors, and channel blockers can now be seen to operate at specific levels within the same cell biological framework (Lynch et al., 2013). Optimistically, we may be approaching a reductionistic (simplifying) conceptual event with regard to enhancing encoding of specific pieces of information. Notably, something of this kind may also be going on for appreciating shared mechanistic impairments present in quite different disorders that interfere with learning: work with a sizable number of rodent models suggests that conditions with disparate etiologies result in a common endpoint failure in cytoskeletal reorganization (Lynch and Gall, 2013).

But there are warning signs with regard to the possibility that the current substrate model may be overly tailored to a specific instance of learning-related plasticity, and in particular to that found in a particular dendritic lamina (stratum radiatum) of a particular hippocampal subfield (CA1). Even within that subfield, there is good evidence that the basal dendritic field exhibits a different form of LTP (Arai et al., 1994; Kramar and Lynch, 2003). And it is now well established that the peculiar mossy fiber connections between dentate gyrus and field CA3 use a form of long lasting potentiation that bears little resemblance to that found in apical field CA1 (Staubli, 1992; Schmitz et al., 2003). It is not unreasonable to expect that additional plasticity variants will be discovered as parametric studies are carried out for other telencephalic connections; e.g., the cortico-striatal glutamatergic synapses (Jia et al., 2010) or the olfactory and associational afferents to piriform cortex (Jung et al., 1990). While these observations greatly complicate predictions about the behavioral effects of putative enhancers, they also offer intriguing possibilities concerning specificity of action. That is, there are reasons to think that different forms of synaptic potentiation may underlie different types, or aspects, of memory. An explicit proposal of this type has been advanced for the basal and apical dendrites of field CA1 (Arai et al., 1994; Kramar and Lynch, 2003): The easily induced, readily erased LTP in the basal dendritic field seems well suited for transient encoding while the higher threshold and more rapidly stabilized form in the apical field is more appropriate for long term memory. An arrangement of this type would be useful in addressing the problem of how to accomplish, through repeated sampling, low noise extraction of constancies from a novel environment (apical dendrites) while at the same time transiently storing a great deal of information much of which can be discarded as being irrelevant (basal dendrites). In any event, testing experimental compounds on various forms of plasticity could lead to agents that target particular forms of memory.

## Cognitive enhancement

### Does augmenting memory enhance cognition?

Memory is such a prominent part of cognition that it seems obvious that enhancing the one will improve the other. However, there may be good computational reasons that cognitive benefit is gained when acquisition is less than optimal in terms of speed and strength. Animals faced with new and complex circumstances need to encode regular features without storing variable, low information elements. Otherwise, as noted earlier, the resultant memories will be noisy and less predictive of future encounters. The spaced trials effect—wherein, temporally separated training trials more efficiently support encoding than does a single “massed” session—can be seen as one adaptation toward better capture of regularities in the learning environment (Hintzman, 1976; Commins et al., 2003; Cepeda et al., 2006). That is, spacing ensures that only elements that are regularly present will be incorporated into memory while transient features will not. An enhancer could obviate the need for spacing by producing strong memory on an initial trial but would be expected to result in a noisy representation.

**Figure 5:**
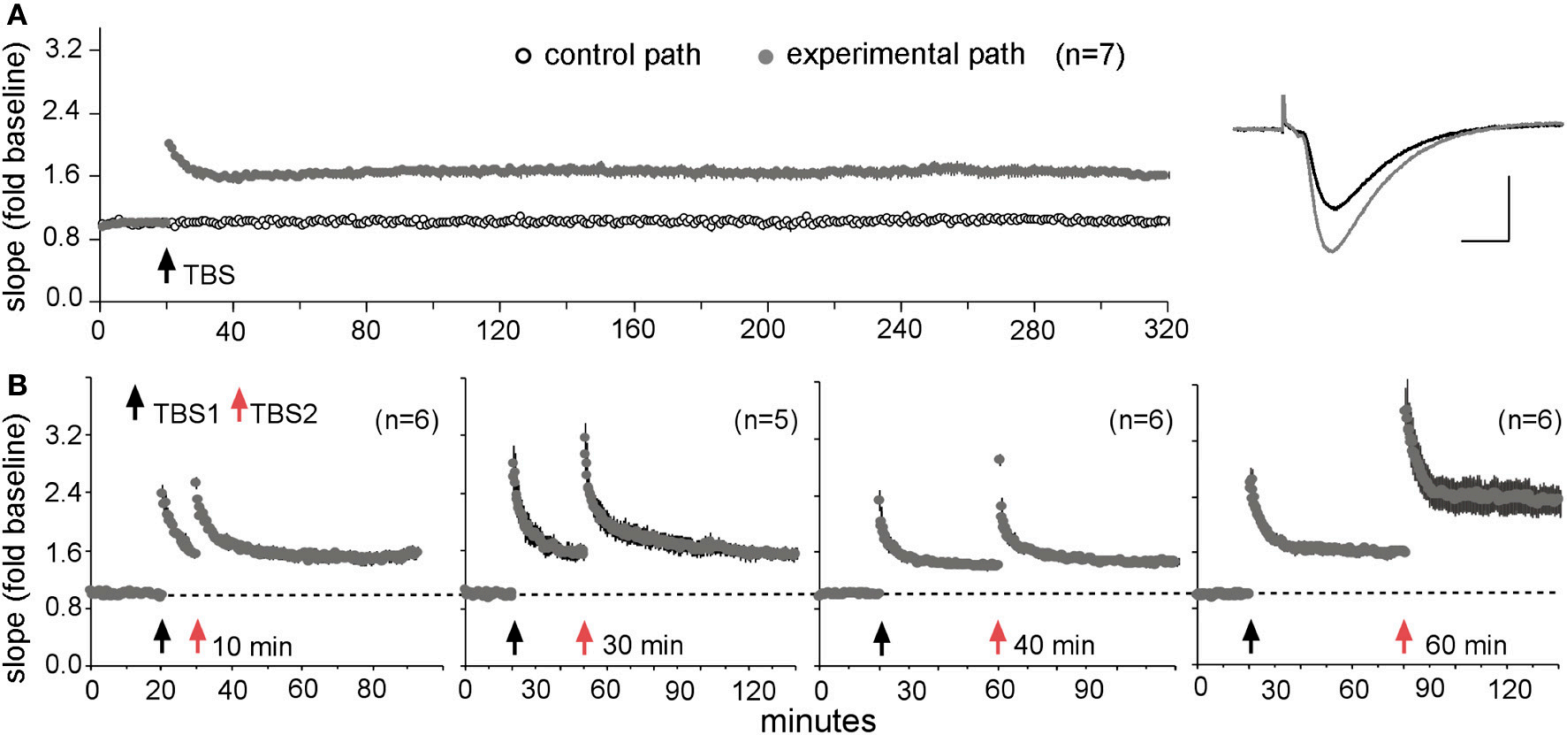
**A “spaced trials effect” for LTP. (A)** Theta burst stimulation (TBS) produces stable LTP. A single train of ten bursts was delivered to one input to the CA1b recording field after 20 min of collecting baseline synaptic responses to 3/min stimulation pulses in adult rat hippocampal slices. A second input to the same dendritic region received 3/min pulses throughout the session. Note that the potentiation of the experimental input did not decay over 5 h of recording (means ± s.e.m.s for seven slices); traces at right show representative baseline (black) and potentiated (gray) responses. **(B)** Effect of a second theta train (TBS2) applied at various times after TBS1. TBS2 produced no further increases in the slopes of the responses when delayed by 10, 30, or 40 min after TBS1, but doubled the magnitude of LTP when applied after a 60 min interval. Modified from Kramar et al. (2012b).

Tests of the above point are lacking but LTP experiments have produced what may be pertinent results. The original descriptions of links between theta burst afferent stimulation and LTP showed that, absent other manipulations, trains of ten bursts produced near maximal potentiation (Larson et al., 1986), a result that led to what has become a standard paradigm. Recently, however, it was found that a second theta train doubles the level of potentiation but only if it is delayed by about 60 min (Figure 5); a third stimulation train produces still more potentiation but only if it is applied at least 60 min after the second. Additional work suggested that this LTP “spaced trials effect” reflects the presence of a large population of synapses with high plasticity thresholds that are “primed” by the first theta episode and then shifted into the potentiated state by the second (Kramar et al., 2012b). These effects fit naturally within the above described substrate map for LTP: activation of synaptic integrins by a first theta burst train was followed by an approximately one hour period before these receptors could be re-engaged by additional stimulation (Babayan et al., 2012). They also set the stage for a first test of how a drug that enhances memory affects a physiological analogue of the spaced trials effect. The results were clear: infusion of an ampakine prior to theta train #1 produced the expected enhancement in the amplitude of LTP1 but also occluded further increases in the level of potentiation following a second, delayed theta train administered in the absence of the drug (Kramar et al., 2012b). Thus, the ampakine enhanced initial encoding (as multiply reported) but did so at the expense of effects of spaced stimulation, and presumably the computational advantages associated with spacing (Lynch and Gall, 2013 for a discussion).

The preceding example describes a situation in which pharmacologically augmenting memory would likely not result in enhanced cognition, at least in complex environments lacking explicit guidelines for effective performance. These are routine circumstances in which demands on cognition are high. But a great deal of cognition involves instances in which significant cues and appropriate responses are salient and predetermined, and irrelevant information is minimized. Under these conditions, enhanced encoding could be of great use in building or expanding cognitive structures. Thus, the effects of memory enhancement on cognition could prove to be situationally dependent with clear benefits in some cases and neutral or even negative influence in others.

### Networks and cognitive enhancement

Discussions of neurobiological processes underlying cognition inevitably begin with the immensely complicated networks formed by cortical neurons, if for no other reason than a lack of realistic alternatives. This fundamental idea suggests two paths to *acute* enhancement. First, improving throughput within established networks should lead to faster computation and better utilization of cognitive time. Second, augmented synaptic communication could allow for the transient assembly of larger than normal networks (e.g., incorporation of additional cortical regions) to deal with a particular problem, and thus the opportunity to execute more complex or even entirely novel computations. In this sense better throughput would add capabilities, perhaps the surest measure of cognitive enhancement. Increased plasticity might add a third route to enhanced cognition by allowing for construction of functional networks that would not likely emerge under normal conditions; however, as noted in the preceding section, positive versions of such effects may be limited to particular circumstances.

There are multiple manipulations that should result in improved throughput. Communication between collections of neurons is greatly improved by synchronizing their activity, something that is accomplished in the cortical telencephalon by system-wide rhythms. These patterns are induced by diffuse ascending projections from the lower brain and drugs that affect these have predictable strong effects on rhythmic activity (Staubli and Xu, 1995; Kowalczyk et al., 2013). But, as mentioned in the discussion of memory, the diffuse systems influence a broad range of brain functions including ones that are vital to survival. And so, as in the case of memory, they do not represent a promising avenue toward enhancement in high functioning individuals. A more likely approach would be to increase transmitter release or post-synaptic responses to transmitter binding at the glutamatergic connections used for the great bulk of intra-cortical communication.

Adenosine, which depresses glutamate release via presynaptic A1 receptors (Dunwiddie and Haas, 1985), is increased in the extracellular environment during repetitive firing by two mechanisms: rapid release from post-synaptic neurons followed by slower release of ATP from glia which is then converted to adenosine by ecto-5′-nucleotidase, an enzyme located on glial membranes (Klyuch et al., 2012; Wall and Dale, 2013). These observations represent a significant part of the tripartite model (terminal bouton, spine, astrocyte) of fast, excitatory transmission (Araque et al., 1999). Selective antagonists of the A1 receptor increase glutamate release in slices and these compounds do indeed reverse impairments in LTP in slices of middle-aged rat hippocampus (Rex et al., 2005). However, despite evidence that the compounds enter the brain (Wall and Dale, 2013), there has been surprisingly little work on *in vivo* effects after peripheral administration. Perhaps the lack of interest with regard to network operations reflects understandable concern about the important roles played by adenosine in the periphery, including actions on the heart and lungs.

Nicotinic receptors for acetylcholine are also found on glutamatergic terminals where they promote release (Wonnacott, 1997) and there is evidence that this increases network throughput (Gioanni et al., 1999). Alpha7-containing and alpha4/beta2 subtypes of the receptors both appear to be effective in this regard (Dickinson et al., 2008). However, the situation is complicated by the likelihood that compounds targeting nicotinic receptors act on cholinergic and GABAergic neurons as well (Wonnacott, 1997; Alkondon and Albuquerque, 2001); moreover, it is not clear that these receptors are present throughout glutamatergic networks. In all, nicotinic receptor agonists and positive allosteric modulators can be assumed to affect portions of excitatory circuitry in the telencephalon while at the same time modifying local processing—via modulation of cholinergic input, interneurons, and glutamatergic collaterals—at individual relays. Net effects will be complex but there is good evidence that the compounds acting on frontal networks enhance “top-down” mechanisms for focusing attention (Sarter et al., 2009). Since pertinent drugs are already in clinical trials (Holmes et al., 2011; Demeter and Sarter, 2013), nicotinic compounds, and especially those targeting the alpha4/beta2 receptor subtype concentrated in brain, have to be seen as one of the most promising of current approaches to cognitive enhancement.

The ampakine compounds described in the earlier section on memory enhancement seem particularly appropriate for improving communication within and between cortical regions. Their mode of action has the virtue of relative simplicity: an extensive body of research from many laboratories has not uncovered any evidence for effects on targets other than AMPA receptors. And they produce the same facilitation of fast, excitatory transmission after peripheral administration as seen with infusions into brain slices. Indeed, ampakines appear to be the only agents so far shown to cause comparable *in vitro*/*in vivo* facilitation of EPSPs. These points lead to two critical experimental questions. First, does increasing monosynaptic transmission result in greater output from a polysynaptic network? This might seem to be a foregone conclusion but each step in a series of neuronal stations has local processing mechanisms (relays are not passive transferal points) dominated by an impressive collection of different types of inhibitory interneurons. These inhibitory elements respond both to inputs directly and to discharges from principle (glutamatergic) neurons; they also form complex local networks among themselves. It is therefore possible that strong inputs are dampened and normalized to a degree such that the second stage of a network may not pass on a larger than normal signal in the presence of an ampakine. Second, assuming augmentation of the signal does occur, what are the functional consequences of enhanced network throughput?

Brain slices provide for the simplest and most compelling tests for circuit behavior because anatomically precise stimulation and recording is possible and extrinsic modulatory (cholinergic, etc.) inputs (cholinergic, serotonergic, etc.) that might influence downstream responses are excluded. Work of this kind has established that weak facilitation of monosynaptic transmission with an ampakine results in a greatly amplified response from the output stage of the trisynaptic intra-hippocampal circuit (Sirvio et al., 1996). These observations accord with the broad idea that facilitated transmission at one connection will lead to a greater number of cells transmitting to the next. Repeated across many stages, each responding to the ampakine, this will produce a multiplier effect for drug action. This argument points to the conclusion that ampakine-type drugs will exert much greater effects in the long chains of glutamatergic neurons that constitute cortical networks than in the much simpler circuits found at lower levels of the neuraxis. Why the multiplier effect doesn't ultimately result in abnormal discharges likely reflects the above mentioned inhibitory interneurons whose influence on projection neurons also grows with increasing glutamatergic drive, as seen in input / output measurements in conventional hippocampal slice experiments. Since inhibition generated by arrival of a glutamatergic input is di- (and multi-) synaptic, there is brief widow in which network facilitation is operative. Sophisticated multi-scale (biophysics, synapses, neurons, and connectivity) computational work indicates the manner in which enhanced throughput can produce useful effects in complex cortical circuits (Bouteiller et al., 2011). However, the effects of increased EPSPs on network responses to rhythmic or complex stimulation are a critical and as yet unstudied issue.

**Figure 6:**
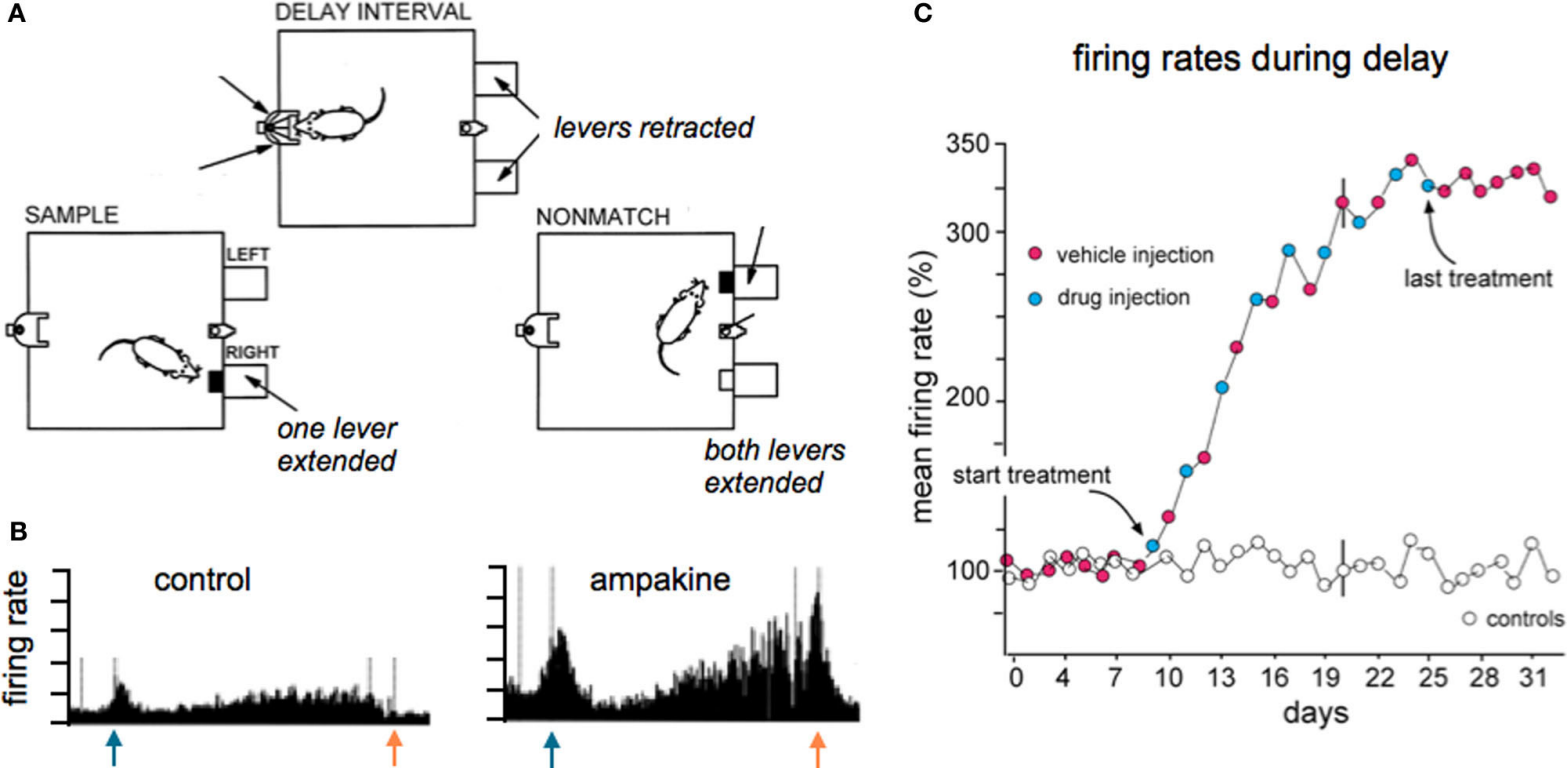
**Ampakine treatment increases neuronal activity in the terminal stage of the intra-hippocampal network and this is accompanied by supra-normal performance on a complex task. (A)** Adult rats were extensively trained in the delayed non-match to sample (DNMS) task, illustrated here, wherein they were required to sequentially (i) press a bar to receive a reward (sample phase; left) at which time a light appeared on the opposite wall of the arena, (ii) move to the wall with the light during the “delay phase” (top) and perform a nose poke for random intervals until the light extinguished, and then (iii) return to the original side of the arena and press the bar that had not been originally selected (non-match phase; right). The level of success in executing the task reached asymptote after weeks of training, after which the animals were separated into vehicle or drug treated groups. Injections were given every other day. Animals given the ampakine prior to individual training sessions showed a progressive increase in correct responses over the next 2+ weeks of daily testing, ultimately reaching levels far higher than the asymptotic level maintained by vehicle-only rats (not shown; see Hampson et al., 1998b). **(B)** Chronic recordings from the same hippocampal neuron collected during task performance prior to and after the start of drug treatment. As shown, the ampakine dramatically increased the firing rate most particularly after the initial response to the sample (blue arrow) and in the delay phase leading up to the non-match choice (red arrow); this pattern and ampakine-related change was typical of field CA1 cells. **(C)** Plot shows the mean firing rate of all units recorded during the delay phase expressed as a percent of the baseline firing rate (prior to ampakine treatment): the progressive increase in firing rate in the experimental rats paralleled the increase in successful trials in the same rats. Rats given vehicle only (open circles) exhibited no change in cell firing or successful task execution over the full period of testing. Note that the increased firing in the drug group was still present on vehicle days (red dots), and indeed for the week following cessation of treatment, an effect that is interpreted as being due to facilitation of LTP-type plasticity on drug days. Adapted from Hampson et al. (1998b).

Evidence for enhanced throughput has also been obtained in studies using *in vivo* analyses of hippocampal projections to frontal cortex (Baumbarger et al., 2001) and chronic recordings from the output stage of hippocampus (Hampson et al., 1998b). The latter rat study showed that the number of cells discharging during key steps in performing a complex task was substantially increased by systemic treatment with an ampakine (Figure 6). Given that the recording site was the terminus of the primary intra-hippocampal circuit, one can reasonably assume that the observed results reflected an augmentation of drug action through a polysynaptic network similar to those described for ampakines infused into hippocampal slices (Sirvio et al., 1996). But the possibility that augmented excitatory drive on ascending biogenic amine systems, whether from the drugs or the behavioral activity they produce, results in generalized increases in neuronal excitability cannot be excluded in these *in vivo* studies.

### Functional effects of enhanced networks

There are relatively straightforward results showing the effects of increasing throughput in cortical networks in simple experimental paradigms. For example, unilateral lesions of the nigro-striatal projections result in a circling response to dopamine agonists; ampakines significantly expand activation of the motor cortex on the side of the lesion and this is associated with a suppression of rotations (Hess et al., 2003). Note that in this case circuits are selectively brought into play that are directly germane to the problem faced by the animal. The expectation of a more subtle version of this effect under conditions in which cortex is performing complex calculations constitutes one basis for hypothesizing that improved network throughput will result in acute enhancement of cognition. It should be noted here that the development of very fast algorithms for extracting core spatio-temporal activity patterns from multi-electrode recordings has made it possible to insert, via stimulation at many sites, information rich patterns to networks in behaving animals. These advances have opened the way to experimental testing of fundamental, long-standing assumptions about how cortical circuits process complex signals from the environment. One recent study of this kind that is particularly germane to the present discussion showed that delivery to CA1 of a “correct” pattern of activation predicted from CA3 recordings during the sample phase of a match to sample problem markedly enhanced performance of monkeys on the subsequent decision phase in difficult versions of the task (Hampson et al., 2013). These dramatic findings encourage the idea that facilitating partial or “weak” network patterns can lead to pronounced improvements in the ability of animals, including primates, to deal with complexity.

As mentioned earlier, enhancement could take the form of acceleration of cognitive activities, and thus allowing for more computations in the same time frame, or expansion of networks and potentially new types of operations. One route for testing the latter possibility would be to overtrain animals to the point at which optimal performance is fully established and then to determine if network facilitation through enhanced transmission allows the subject to go beyond normal limits. There is very little work of this kind for ampakines or any other putative enhancer but suggestive results have been described. The study noted above in which ampakines expanded the hippocampal response during learning (Figure 6) also found that overtrained rats significantly improved their learning scores under the influence of the drug. Remarkably, the animals then continued to perform at supra-normal levels in the absence of the ampakine. Detailed analyses showed that the animals shifted response strategies in a manner that reduced proactive interference between trials (Hampson et al., 1998a). In essence, the drugs opened the way to expanded networks and the development of higher order rules that cannot otherwise be acquired even with weeks of training. Another example of going well beyond normal limits has been described for monkeys performing a challenging delayed match-to-sample problem (Porrino et al., 2005). The animals were trained to asymptote to identify, as indicated by movement of a computer cursor, a previously seen real world cue from a group of similar objects. Performance on the task was increased dramatically with ampakine pretreatment. Brain imaging studies then uncovered a remarkable result: the ampakine intensified activity in frontal and temporal cortices but also led to the engagement of a superior parietal region, the precuneus, which was inactive during vehicle trials. The precuneus is thought to be critical for envisioning future actions by humans. In any case, these results in a primate provide an example in which expansion of cortical networks is associated with a lifting of limits on performance in a cognitively demanding problem.

These few studies using overtrained animals, exciting though the findings may be, are hardly sufficient to establish the general point that increases in network throughput result in beyond normal performance on challenging problems. Experiments of this type are not common because they involve major investments in time and technology. And it will be noted that they focus on problems that are sharply defined with regard to cues and appropriate responses. One can fairly ask if these conditions capture the essence of cognition as a free flowing processing of the enormous complexities generated by the exterior and interior worlds of humans. This point is picked up in the following section.

### Future neurobiological studies on cognitive enhancement

A question running through this review, and alluded to immediately above, concerns the extent to which we can consider problem solving by animals as a fair descriptor of cognition. One can hardly question the proposition that the analyses of different computations performed by distinct frontal subfields in rats (e.g., credit assignment to particular serial actions, set shifting, focusing of attention; Turner et al., 2004; Sugrue et al., 2005; Demeter and Sarter, 2013) will provide deep insights into how humans resolve real world issues. But here we encounter the problem of how to define cognition and whether or not it can be understood in simple computational terms. To be specific, what might be needed are studies testing whether putative enhancers improve the performance of sophisticated (highly experienced) subjects dealing with novel circumstances of great complexity and without the benefit of external supervision. If nothing else, this would bring experiments closer to the human condition and thereby help explain why animal studies on cognition and memory have such a poor record in predicting human outcomes.

**Figure 7:**
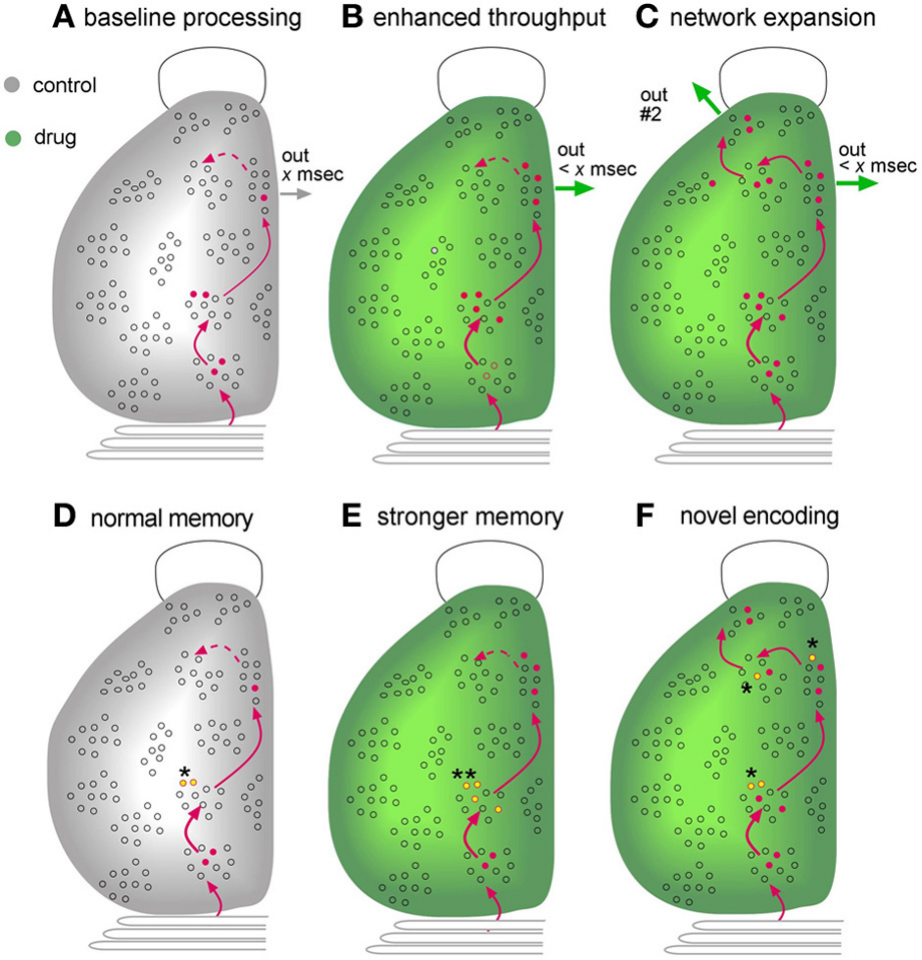
**Mapping as an endpoint measure for evaluating the effects of putative enhancers**. Schematic illustration of a dorsal view of the left side of a rodent brain, with an open outline of the olfactory bulb at top and the cerebellum at bottom. Gray is for control subjects while green denotes results obtained in the presence of an experimental treatment. **(A–C)** Network Mapping. The figures describe the distribution of cells activated during learning of a novel environment, as could be determined from maps assembled from serial sections labeled for activation of Immediate-Early Genes (see text). Combining this information with neuroanatomy (red lines) would provide a picture of the extent to which known circuits were engaged during learning. **(A)** Interaction with the environment increases the number of cells firing (red dots) in three successive stages of a transcortical network, resulting in output from the third stage. A fourth region that that is weakly innervated by preceding network stages (dotted line) is not engaged. **(B)** A compound that facilitates excitatory synaptic transmission increases the number of neurons that respond to the input and thereby reduces circuit-throughput time (< *x* ms) and generates a more robust network output. **(C)** An additional possibility, supported by experimental work, is that improved transmission adds regions to the network, resulting in novel outputs (“out #2”). Potentially, the cortex in this instance would gain new capabilities for dealing with complex problems. **(D–F)** Mapping of encoding sites (engrams). Recent advances have made it possible to identify synapses that are modified by recent learning. Mapping studies for hippocampus strongly suggest that such effects are restricted to a small number of sites in a multiple stage network (see text). **(D)** Under control conditions, memory-related synaptic changes, such as LTP, are restricted to stage #2 (yellow dots, asterisk) of one network engaged during learning. **(E)** An enhancer could increase the number of neurons on which such synaptic changes occur in regions in which they are normally found (double asterisks). Such an effect is predicted to result in the formation of stronger memory (e.g., more resistant to extinction). **(F)** Improved transmission relating to an experimental treatment reduces the threshold for inducing memory-related synaptic alterations such as LTP. It follows from this that the manipulation could result in synaptic modifications in regions at which they do not normally occur (multiple asterisks). This is predicted to produce an elaborated, novel representation of the learned material.

Much of the present discussion centered on the proposition that enhancing network throughput will have positive effects on cognition (Figures 7A,B). It will be recognized that most of the material presented in support of this idea dealt with specific neuronal circuits or opportunistic discoveries of network expansion. A more systematic, agnostic description of how experimental compounds affect the vast number of forebrain circuits is badly needed. This could be obtained using activity-regulated immediate early gene expression to provide an index of the recent history of neuronal firing. Such analyses would provide a picture of the networks assembled to deal with complex circumstances, surely an initial step toward a mechanism based theory of cognitive operations, and add an information rich step for the screening of experimental compounds. There is also the possibility that network maps of drug effects would be predictive: measures of intensified activity within, or expansion of, behaviorally engaged circuits (Figure 7C) should lead to explicit hypotheses about the origins of psychological changes.

Another type of mapping may also prove useful in future searches for cognitive enhancers (Figures 7D–F). As noted, changes in the numbers of individual synapses associated with LTP-related actin regulatory proteins have been detected in hippocampus following learning. Notably, the labeled synapses were larger than their neighbors (Fedulov et al., 2007), an effect that was also seen with LTP (Chen et al., 2007). Continuing advances in the technology for identifying synapses engaged in plastic changes that underlie learning have made it possible to plot the distribution, across entire cross-sections of the hippocampus, of subfields that reliably contain many such contacts. A first study using the mapping method to plot synapses undergoing plasticity following learning of a new environment detected only three out of forty-two sampling zones within hippocampus that match this description; imposing a response contingency that interfered with free exploration eliminated the effect (Cox et al., 2014). These results cannot be taken as indicating that learning related synaptic adjustments only occur within these three sites; it is entirely possible that such effects are present in many regions but vary between subjects and/or are not numerically large enough to be detected with current procedures. But the results strongly suggest that encoding of one type of spatial information is not homogeneously distributed but instead occurs at high levels in a surprisingly small number of locations. In essence, they constitute a first, albeit crude memory map that covers one septo-temporal segment of the hippocampus.

Localizing memory has been a much discussed topic among brain scientists since the early days of the last century; subsequent attempts to map the distribution of encoding sites acquired an evocative title: “The search for the engram” (Thompson et al., 1976; Thompson and Krupa, 1994). Maps, or engrams, are of evident importance to the development of neurobiological theories of how memories are recalled but they are also of potential significance with regard to network events related to cognitive enhancement. The discussion to this point has stressed the effects of transiently facilitating network throughput but it is noteworthy that certain of the manipulations suggested for this purpose, such as increasing neurotrophic factor signaling, may also promote stable changes in connectivity. But would such changes simply increase the efficacy (e.g., throughput time) of extant circuits or would they allow for the emergence of new and persistent networks? This distinction returns to the earlier consideration of enhancement as reflecting faster processing or the introduction of new capabilities. The technology for mapping the location of learning-driven synaptic modifications may allow for neurobiological, as opposed to purely behavioral, tests of the question: does an experimental manipulation intensify the map (more synapses with LTP-related changes at normal sites) vs. creating additional regions with high numbers of modified connections (Figures 7D–F).

In all, a futuristic combination of network mapping with localization of synaptic changes could shift the evaluation of putative enhancers from exclusively behavioral endpoints to the presumed network substrates of cognitive operations. Such a step would likely lead to the many advantages in problem conceptualization historically associated with reductionism in science.

### Conflict of interest statement

The authors declare that the research was conducted in the absence of any commercial or financial relationships that could be construed as a potential conflict of interest.
